# Toxic Stress: Effects, Prevention and Treatment

**DOI:** 10.3390/children1030390

**Published:** 2014-11-03

**Authors:** Hillary A. Franke

**Affiliations:** Department of Pediatrics, Section of Pediatric Critical Care, University of Arizona , Tucson, AZ 85716, USA; E-Mail: hfranke@peds.arizona.edu; Tel.: +1-520-626-5485; Fax: +1-520-626-6571

**Keywords:** toxic stress, early childhood adversity, resilience, relaxation response, pediatrics

## Abstract

Children who experience early life toxic stress are at risk of long-term adverse health effects that may not manifest until adulthood. This article briefly summarizes the findings in recent studies on toxic stress and childhood adversity following the publication of the American Academy of Pediatrics (AAP) Policy Report on the effects of toxic stress. A review of toxic stress and its effects is described, including factors of vulnerability, resilience, and the relaxation response. An integrative approach to the prevention and treatment of toxic stress necessitates individual, community and national focus.

## 1. Introduction

Stress is a term commonly used to describe the response to the demands encountered on a daily basis throughout one’s lifetime, and it is related to both positive experiences and negative experiences. Stressors are agents that produce stress. Stressors may be physical, emotional, environmental or theoretical, and all may equally affect the body’s stress response. The stress response, also known as the “fight or flight” response or general adaptation syndrome, comprises the physiologic changes that occur with any encounter of stress or perceived stress to the individual [1,2]. This stress response is a result of stimulation of the sympathetic nervous system resulting in a cascade of neuro-endocrine-immune responses, with reproducible physiologic effects that include an increase in respirations, heart rate, blood pressure, and overall oxygen consumption [3,4]. In most situations, the physiologic changes associated with the stress response are transient, with the body returning to its baseline state when the stressor is removed. Toxic stress responses include a prolonged or permanent abnormal physiologic response to a stressor with risk of end organ dysfunction [5,6]. Toxic stress can affect anyone, and children are no exception.

Childhood toxic stress is severe, prolonged, or repetitive adversity with a lack of the necessary nurturance or support of a caregiver to prevent an abnormal stress response [5]. This abnormal stress response consists of a derangement of the neuro-endocrine-immune response resulting in prolonged cortisol activation and a persistent inflammatory state, with failure of the body to normalize these changes after the stressor is removed [6,7]. Children who experience early life toxic stress are at risk of long-term adverse health effects that may not manifest until adulthood. These adverse health effects include maladaptive coping skills, poor stress management, unhealthy lifestyles, mental illness and physical disease [5,6,8,9,10,11,12,13].

In January 2012, the AAP published a policy statement on the effects of toxic stress and childhood adversity using an ecobiodevelopmental framework to approach this important public health concern [8]. This framework acknowledges the interactions of personal experiences, environmental influences, and genetic predispositions that shape the learning and behavior of an individual across his or her lifespan. The AAP put out a call to action, including the following recommendations: a need for understanding that lifelong disparities are determined by social, behavioral and economic determinants; proper training of all healthcare providers in the effects of toxic stress; and ongoing advocacy by pediatric providers. The medical home becomes an important venue for thorough anticipatory guidance, active screening for at-risk children, knowledge of local resources, identification and implementation of interventions to decrease sources of toxic stress, and development of comprehensive treatment plans for mitigation of toxic stress effects [8,9].

## 2. Stress—Positive, Tolerable, Toxic

Early childhood experiences play a large role in how the brain develops and functions. Interactions with the child and his or her environment affect long-term learning, behavior, and health. Healthy brain architecture relies on responsive caregivers and positive relationships that help children learn to handle stressful experiences. In general, the stress response is a physiologic response to an adverse event or demanding circumstance and includes biochemical changes to the neurologic, endocrine, and immune systems.

### 2.1. Positive Stress

A positive stress response is a normal stress response and is essential for the growth and development of a child. Positive stress responses are infrequent, short-lived, and mild. The child is supported through this stressful event with strong social and emotional buffers such as reassurance and parental protection. The child gains motivation and resilience from every positive stress response, and the biochemical reactions that occur with such a stressful event return to baseline [5]. Examples include meeting new people or learning a new task.

### 2.2. Tolerable Stress

Tolerable stress responses are more severe, frequent or sustained. The body responds to a greater degree, and these biochemical responses have the potential to negatively affect brain architecture. Examples include divorce or the death of a loved one. In tolerable stress responses, once the adversity is removed, the brain and organs recover fully given the condition that the child is protected with responsive relationships and strong social and emotional support.

### 2.3. Toxic Stress

Toxic stress results in prolonged activation of the stress response, with a failure of the body to recover fully. It differs from a normal stress response in that there is a lack of caregiver support, reassurance, or emotional attachments. The insufficient caretaker support prevents the buffering of the stress response or the return of the body to baseline function. Examples of toxic stress include abuse, neglect, extreme poverty, violence, household dysfunction, and food scarcity. Caretakers with substance abuse or mental health conditions also predispose a child to a toxic stress response. Exposure to less severe yet chronic, ongoing daily stressors can also be toxic to children [14]. Early life toxic stressors increase one’s vulnerability to maladaptive health outcomes such as an unhealthy lifestyle, socioeconomic inequity, and poor health; however, these stressors do not solely predict or determine an adult’s behavior or health [10,14].

## 3. Toxic Stress Effects

A child experiencing adversity is at risk of permanent changes to brain architecture, epigenetic alteration, and modified gene function. Implications for long-term health and developmental effects are critical, including increased risk for stress-related diseases [5,15]. The toxic stress response affects the neuroendocrine-immune network, and the response leads to a prolonged and abnormal cortisol response [6,7]. The resultant immune dysregulation, including a persistent inflammatory state, increases the risk and frequency of infections in children [16,17,18]. The toxic stress response is believed to play a role in the pathophysiology of depressive disorders, behavioral dysregulation, PTSD, and psychosis [13,19,20,21,22,23,24,25]. Adults who endured early childhood adversity also experience more physical illness and poor health outcomes [11,16,17,18,26,27]. These poor health outcomes are varied and include alcoholism, chronic obstructive pulmonary disease, depression, cancer, obesity, increase in suicide attempts, ischemic heart disease and myriad other disease processes [11,28,29]. A proposed model relating the effects of toxic stress to a potential increased risk of cancer suggest both a direct effect of stress on biological systems and an indirect effect of poor health behaviors as responses to stress [30].

Large longitudinal studies like the Adverse Childhood Experiences (ACE) study and the Coronary Artery Risk Development in Young Adults study show correlation with the number of ACE and the increased risk of multisystem health problems in a graded fashion [11,31]. Notably, low levels of parental warmth and affection with high levels of abuse had the highest multisystem health risk as adults. Inversely, strong parental warmth and affection during childhood was associated with less health risk in adulthood [31]. Maternal warmth appears to buffer toxic stressors, such as growing up in extreme poverty [31,32]. Maternal support may have a protective effect on childhood abuse, and it also appears to be a variable determining a positive response to therapies [33,34]. Ongoing familial support including maternal care and paternal protection have been shown to affect treatment response in situations of abuse and are more predictive of success than the type of the abuse experienced [33].

## 4. Resilience and Vulnerability

Resilience is the ability to properly adapt to adversity despite the conditions. It is dynamic and plays a large role in a toxic stress response. Not all individuals who experience repeated childhood adversity experience poor health, and resilience may provide a buffer. Resilience factors are numerous and change over time in an individual. Children with resilience have been identified as having the following characteristics: higher IQ, easy temperament, a perception of competence, a positive self concept, a realistic sense of control of the situation, empathy, and social problem solving skills [34,35,36,37]. Factors that predict resilience in children experiencing adversity include a solid relationship between the child and parent as well as a high quality relationship between the child and teacher [37]. Additional resilience factors identified include adequate social support, marriage quality, the physical and mental health of the parent, and the parent’s sense of efficacy [33,34,37,38,39]. Prior adaptive behaviors that result in overcoming adversity strengthen resilience. Focus on early interventions to strengthen resilience factors may help to minimize a toxic stress response [28].

Factors believed to increase vulnerability to early toxic stress include an individual’s increased sensitivity to both psychological and physiologic stress with a decreased resources for social and psychological support to help with stress coping skills [18]. Other sources of vulnerability may include poor social support, developmental delays, abusive parenting, and maladaptive behaviors in response to adversity [36].

## 5. Relaxation Response

Documented in the 1970s by Herbert Benson, the relaxation response is a state of decreased sympathetic nervous system activity that opposes the stress response [4,40]. Physiologic effects of the relaxation response include a reduction in respirations, heart rate, blood pressure, and oxygen consumption, with an increase in heart rate variability; these effects have been elicited regularly and repetitively with techniques including transcendental meditation, autogenic hypnosis, Zen and yoga, contention, sentic cycles, and progressive muscle relaxation [40]. Conscious effort and practice are indicated for achieving ongoing effects of the relaxation response, however many techniques require minimal instruction or practice to immediately produce the calming effects.

Techniques may be as simple and informal as repeating a word or phrase while sitting comfortably in a quiet area or taking several deep breaths [40,41]. Formal programs such as The Relaxation Response Resiliency Program (3RP) use adaptive coping mechanisms for chronic stress [42]. The 3RP uses components of a relaxation response strategy, stress coping, growth enhancement and inter-connectedness to address and promote resiliency, and this regimented approach may demonstrate benefit for individuals in communities with chronic stress [42]. Other techniques, such as biofeedback, guided imagery, and mindful awareness may take time to establish into routine. While techniques vary, there are four basic components that, when combined, produce the relaxation response; these include a repetitive sound or phrase, a passive attitude of disregard to distraction, relaxed positioning, and a quiet environment [40]. There are many ways to reach a state of relaxation response, and for best achievability and sustainability, the individual’s preference to technique and resources such as time and financial investment for the technique must be taken into account.

The relaxation response has been effective as a tool for situations in which excessive sympathetic activity prevails, such as would be considered in exposures to toxic stress [40]. Children, their parents, family members and community members may benefit from awareness of the relaxation response, and these skills may help build resiliency for future stressors.

## 6. Unique Role of the Pediatrician to Address Toxic Stress

A pediatrician-led medical home has been identified by the AAP as an important venue to best identify risk factors, to prevent and reduce toxic stress, as well as to build resiliency in individuals and families [8]. Ideally, the pediatrician’s perspective of childhood health that places focus on prevention, use of developmental milestones, and advocacy for a safe childhood experience makes for a broad base upon which to address toxic stress. Realistically, pediatricians have ever-increasing demands with decreasing time and resources, making this additional screening and treatment difficult, if not an impossible challenge. How does a pediatrician address these important needs for their patients and families? The pediatrician-led medical home requires resources in order to adequately provide standard of care and to also be able to meet the needs of individuals, family, and community with regards to toxic stress. This challenge to provide the care required for our nation, communities, and individuals does not have a simple answer. Programs in place that are successful will build awareness of need for programs throughout the country.

Some useful interventions as well as hardwired processes in the pediatrician’s office may address toxic stress while not causing an enormous strain on already limited resources. For example, prioritizing the hardwiring of a toxic stress screening process upon entry of a patient to the exam room may be helpful. Posting handouts on instructions for breathing techniques, a list of free smart phone applications on biofeedback, or websites for stress reduction may give patients and families an awareness of relaxation. Some topics may be discussed during certain well child visits if time permits. A monitor in the waiting area with topics of childhood health may include toxic stress as a topic. Pediatricians may start with simple steps and as each technique is hardwired one may consider tackling another.

## 7. Screening for Toxic Stress

A child risks maladaptive stress responses when exposed to childhood adversity and toxic stress. The first several years of life are sensitive periods of time for increased neuroplasticity, after which it begins to wane [43,44]. If primary preventive measures are implemented during the early, sensitive windows of development, appropriate stress responses to adversity may result. Screening is a means to identify those children who would benefit from both preventive measures and, if need be, therapeutic interventions.

Factors that place a child at risk of maltreatment overlap those with risk of toxic stress, and the AAP recommends screening for factors such as social isolation, poverty, unemployment, low educational achievement, single-parent home, non-biologically related male living in the home, and family or intimate partner violence, young parental age, and parent factors such as low self-esteem, substance abuse, and depression [45]. Protective factors for child maltreatment may also be useful to attenuate a toxic stress risk, and some of these factors include presence of a caring and supportive adult, positive family changes, structured school environment, access to healthcare and social services, involvement with religious community or extracurricular organized activity [45]. The AAP has not as of yet identified a specific screening tool to be used for toxic stress or one to be incorporated within usual screening protocols such as the developmental milestones. Social-emotional screening has been shown to predict behavioral problems and would fit with the need to identify children at risk of toxic stress [46]. Promise has been seen with use of the Ages and Stages Questionnaires: Social-Emotional screening tool, however broad use within multiple pediatric settings is necessary [47].

## 8. Prevention of Toxic Stress

Toxic stress is a function of the absence of buffers to return the stress response to baseline, and it is important to consider preventive measures that promote positive environmental influences and interactions with supportive caretakers. Routine anticipatory guidance, which encourages positive parenting, strengthens support for families, and builds resilience, helps develop the buffers required to handle stress and avoid toxic stress. AAP resources such as *Bright Futures, Connected Kids, Medical Home* and “The Pediatrician’s Role in Child Maltreatment Prevention” offer recommendations [45,48]. Preventive interventions should be focused on only those children at risk of adverse childhood events. Children screened and found to have no risk of toxic stress may actually experience a detrimental stress response to an intervention [37]. Preventive measures to improve resilience in the child are notable, as is focus on aiding assisting the caretaker. Targeting the caretaker’s stressors and improving the caretaker’s capacity to provide safe, stable and nurturing relationships may mitigate any toxic stress response in children [31,32].

## 9. Treatment of Toxic Stress

An integrative approach to manage and prevent stress in general may play a vital role in preventing and treating childhood toxic stress. Treatment of toxic stress requires timely intervention, and goals are to decrease stressors and the individual’s response to stressors, to minimize vulnerability, and to strengthen resiliency. Treatment should be aimed at the needs of each individual. Toxic stress effects are widespread and involve community and implicate healthcare on a national level. Approach to the individual, the caretakers and immediate family, the community, and awareness at the national level are indicated.

### 9.1. Individual and Family

Helping children learn to shut off their stress response in a healthy manner is important, and multiple approaches can be used for this goal. Conventional approaches such as referral to social work, psychology, or psychiatry may be beneficial, however these interventions can be costly. If therapy is indicated, the type of therapy depends on the adversity experienced. Evidence supports the use of parent-child interaction therapy, child-parent psychotherapy, cognitive behavioral therapy, and trauma-focused psychotherapy for children showing signs of toxic stress [33,35].

Integrative approaches to the child include attenuating the stress response and building resiliency. Tapping into the relaxation response with breathing techniques, guided imagery, and biofeedback may be well received depending on the age of child and if different techniques are offered as options. Mindfulness-based stress reduction or mindfulness-based cognitive therapy are time-intensive therapies requiring an instructor with years of experience, but if available and feasible, may be beneficial as studies suggest decreased anxiety, improved mood, relief of psychological distress and strengthened wellbeing [49]. Biofeedback is an effective tool to decrease heart rate and respiratory rate, increase heart rate variability and improve pulmonary peak flows [50,51]. Patients are able to measure changes objectively, which may benefit those with a suspicion for integrative approaches. Patients with comorbidities such as asthma may profit from this relaxation modality by learning about their body’s response to different input while also gaining the control to potentially halt exacerbations of illness. Other mind-body interventions shown to decrease stress include hypnosis, guided imagery, music therapy, and progressive muscle relaxation [52,53,54,55,56,57,58].

A systematic review of cortisol regulation in children demonstrated numerous interventions to decrease stress response. Interventions centered on the child or adolescent showing benefit included guided imagery, social and educational enrichment, and in-home sessions to develop language skills [59]. Interestingly, improved cortisol response was also seen in children when interventions focused solely on the child’s caretaker [59]. Parenting classes, home visits to improve parenting practice, telephone support, family-based programs, access to social resources for parents, problem solving and information seeking skills, and peer support were beneficial [59]. A focus on the caretaker may be one part of the approach to reduce toxic stress risk. Having stable relationships so a child senses a safe environment is important to avoid toxic stress effects [60].

Teaching families these techniques for relaxation takes time and in a busy practice it may be difficult to accomplish. Using handouts and giving parents online resources may help spread information about the importance of stress reduction and give step-by-step instructions. Judicious use of the internet may supplement the pediatrician’s current resources with further information including techniques to help build resiliency, to teach parents to establish healthy connections, and to identify tools to cope with chronic stressors. For example, helpguide.org addresses many topics including stress reduction, parenting and attachment, relationships, and child issues [61]. The American Institute of Stress has information and techniques for dealing with stressors in daily life, the workplace, and in certain family situations such as those in the military [62]. Biofeedback products are available online, with information for research studies available as well [63,64]. Mind-body topics are also available on medical websites and websites of integrative medicine organizations. Relaxation response techniques are being studied in the virtual environment, and individuals report that having an ability to personalize the virtual online environment as well as participate anonymously without judgment has been well received, and it is anticipated that future online experiences with mind-body medicine education will expand [65]. Numerous stress reduction applications for smart phones have been developed and many are free for download. Internet accessibility may not be available for all families in a pediatric practice, however summary handouts or referral to a public library may be feasible.

### 9.2. Community

Community-based interventions that strengthen neighborhood-level resources may be most effective in buffering the toxic stress response in children [60]. It is important to know what community resources, outreach programs, and active volunteer groups are in the area for your patients in which to become involved or if they could benefit from additional services. Groups may be willing to volunteer time and resources to use to improve the lives of children, only they may not be aware of this important issue. Looking to these groups and initiating contact about important pediatric topics may lead to change, and at the least will increase community awareness of toxic stress.

Positive environmental changes can improve childhood outcomes, even in extreme cases of adversity. Community based interventions have been shown to be effective and long-term follow up of children involved in interventional programs exhibit enduring behavior and health effects [28,66,67]. Early-intervention programs such as Head Start may affect a child’s development and exposure to positive experiences while decreasing the adversity of hunger that many children would otherwise experience [6,44].

Community organized home visits may be a mechanism to lend support to caretakers in the natural home environment [28]. Caretaker skill building, including improving the caretaker’s employability and resultant economic stability, is imperative for protecting children from toxic stress [60].

### 9.3. National

Early toxic stress affects our entire nation. The effects of early toxic stress are realized through adulthood, with large costs to the individual as well as to society. The potential exists to prevent adult-onset diseases by targeting and promoting healthy stress responses in childhood. National awareness of the effects of adverse childhood events allows further opportunity for interventions. Pediatricians are asked to involve schools, community, and government to help aid with toxic stress interventions [28]. Advocacy on a national level is imperative to lobby for funding of meaningful programs and to gain support, financially and otherwise for pediatric healthcare workers to appropriately and adequately screen for toxic stress in the office setting. The AAP is present at the national level and will continue to advocate for the better care of children, including awareness, prevention, and early treatment of toxic stress.

## 10. Conclusions

Toxic stress burdens society, and everyone is susceptible to its effects. Awareness of early childhood adversity risk and resultant downstream effects of toxic stress is key. Prevention must begin early with the targeting of at-risk populations. Protection of children from toxic stress requires a multi-faceted approach, including interventions that will target the child, the caretaker, and the environment. Strengthening the stability of the family as well as the community affords environmental protection against childhood effects of toxic stress. Use of proven stress reduction strategies is important, and many mind-body approaches are effective in eliciting the relaxation response. Appropriate management of the adolescent or adult patient with resultant health effects from toxic stress necessitates full knowledge of the long-term effects of the toxic stress response, including the need for stress reduction, coping techniques, and use of an integrative approach to therapy.
